# Location, location, location: Close ties among older continuing care retirement community residents

**DOI:** 10.1371/journal.pone.0225554

**Published:** 2019-11-25

**Authors:** Liat Ayalon, Inbal Yahav

**Affiliations:** 1 Louis and Gabi Weisfeld School of Social Work, Bar Ilan University, Israel; 2 Graduate School of Business Administration, Bar Ilan University, Israel; Graduate School of Public Health and Health Policy, City University of New York, UNITED STATES

## Abstract

This study examines two theoretical explanations for the existence of close ties among continuing care retirement community residents: the attractiveness theory, which suggests that residents who possess certain attributes are more likely to be perceived as appealing to others; and the homophily theory, which argues that individuals are more likely to have close ties with people who share similar attributes. As a variant of the homophily theory, we also examined whether sharing a physical location makes the existence of certain connections more likely. Data from four continuing care retirement communities were used. To test the attractiveness theory, correlations between the number of individuals who named a person as a significant contact (ego’s in-degree) and ego attributes were examined. To test the homophily theory, the median value of existing ties was compared against all possible social ties as though they were randomly formed. Finally, to further test the role of the institutional culture against various motivations that drive social ties—attractiveness and homophily—we used link prediction models with random forests. In support of the homophily theory, beyond the institutional culture, the only consistent predictor of the existence of close ties among residents was sharing a wing in the retirement community (geographic proximity). Therefore, we discuss the role of the physical location in the lives of older adults.

## Introduction

Older adults often report high levels of aloneness and loneliness [1, 2]. In response to the unique social needs of older adults, there are a variety of formal (paid) outlets, which specifically aim to provide older adults with social opportunities [3, 4]. One such outlet, which actively attempts to alleviate loneliness among older adults, is the continuing care retirement community (CCRC) [5].

In Israel, where the present study took place, CCRCs are privately funded and are considered an expensive residential alternative available only to a select few [6]. The setting is marketed as an opportunity to celebrate “old age” [7]. It is available to older adults who are independent upon entrance and offers a variety of social and health services to cater to the interests and needs of the residents [8]. Because the CCRC is considered the “last stop,” assisted living services are often available on the premises, with no need for the older adults to relocate, so that they can enjoy continuity. The nursing unit is also located on-site, but is usually somewhat remote from the independent unit [9].

Older adults often report that loneliness and social isolation are among the main reasons for their relocation into a CCRC [10]. Enhanced social opportunities and contact with other residents are identified as important “pull” factors responsible for attracting older residents to the CCRC [10, 11]. Following relocation to a CCRC, older adults tend to report a reduction in loneliness and an improvement in their social ties with residents and friends [12, 13]. Although researchers have argued that the number of ties between individuals who make up the social network in CCRCs is quite sparse, with most residents not even knowing each other [14], others have shown that the CCRC is effective in alleviating loneliness and facilitating social connections among its residents [15, 16]. However, some research has stressed the presence of cliques, conflicts, and loneliness in residential care settings [17, 18]. Moreover, a recent review of the literature has concluded that the rates of loneliness in long-term care are high, with more than 50% of the residents reporting loneliness [19]. However, this review encompassed a large number of long-term care settings, including nursing homes and nursing units, which involve older people who might be experiencing substantial physical and/or cognitive impairments.

### What predicts the presence of close social ties?

This study evaluated potential predictors of the presence of close social ties between CCRC residents. This is important because of the increasing popularity of CCRCs as a residential alternative for older adults. This is also important given the significance of close ties, defined in this study as relationships that allow people to comfortably reveal their intimate secrets and thoughts to others, in the lives of older adults [20]. We capitalize on two major theoretical bases, outlined below.

The first theory, referred to as the attractiveness theory, argues that certain individuals are socially more attractive because they have certain characteristics that make them so [21]. For instance, we know that physical appearance, extraversion, and self-confidence serve as magnets and make some people more socially attractive in the eyes of others [22–24]. In the CCRC, good health could be considered a desired commodity, given its relative scarcity and high value for residents [25]. In support of this claim, past research has shown that health is a potential asset that makes CCRC residents more socially attractive [26]. Consistently, a higher level of cognitive functioning has also been shown to be associated with a larger social network [27, 28]. These findings obtained from an analysis of the entire social network of CCRC residents are supported by qualitative research that has identified physical and cognitive functioning as valuable assets of CCRC residents [9, 25, 29].

Another theoretical basis that could potentially account for the presence of social ties between CCRC residents is homophily [30]. Homophily—“birds of a feather flocking together”—is when people form closer ties to those with whom they have characteristics in common [31]. There is ample research to show that homophily exists with regard to status attributes (e.g., gender, race, education, and age), for instance [31, 32].

Researchers have argued that the most common basis for the development of social ties based on homophily is race, followed by other characteristics such as age and education [31]. Specifically, racial segregation takes place even in mixed-race schools and neighborhoods, and people of different racial groups are less likely to form close relationships with each other [33, 34]. Age has also been found to be a very strong indicator of homophily, as our entire social lives are organized around it [35]. In later life, age segregation might be particularly pronounced [36] and even intensified by older adults’ living arrangements [29]. Education is another well-known basis for the development of social ties, with individuals of similar socioeconomic status grouping [37]. This tendency might be intensified by the fact that individuals tend to choose their living environment in relation to their socioeconomic status [38].

Another very consistent predictor of social ties is physical location [39–41], following the assumption that two individuals who are physically in close proximity exhibit greater geographic homophily [31]. A recent study conducted in a CCRC showed that older adults who shared the same hallway were more likely to like each other [42]. This study builds on a very large body of literature, dating back to 1950, that shows that physical proximity brings people not only physically but also emotionally closer together [43]. Others have found geographic proximity to be a consistent predictor of friendship formation between school-age children as well as adults [44–47].

Homophily also explains the potential role of the institutional culture in the formation of social ties. “Institutional culture” refers to the values, customs, attitudes, and behaviors that are shared collectively among the individuals who make up the institution, such as the CCRC in the present study, and go beyond the individual characteristics of the residents [48, 49]. It is possible that some individuals choose certain institutions because of their high degree of similarity with its members in other qualities, such as age, gender, or education. Alternatively, individuals may become more similar over time, simply because they belong to the same institution.

The institutional culture matters because it can shape our likes and dislikes [50]. The institutional culture also helps shape our communication style [51, 52], the type of relations formed, and the strength of relationships [53]. Consistently, individuals may select certain institutions and not others because of already existing similarities among members. Although institutional culture can be seen as a case of homophily, it differs from the other forms of homophily tested in this study. Institutional culture defines the entire social network in each CCRC rather than homophily at the dyadic level (ego-alter relations), which is the case of all other characteristics examined in this study, such as health and age.

### The present study

A unique advantage of the present study is its multi-site nature. In contrast to past research [42, 54], this study examines four different CCRCs. Hence, it assesses the generalizability of the findings beyond the characteristics of the unique setting. By examining four different settings, our study adds to a growing body of research that has argued for the importance of integrating the concept of institutional culture into the field of social networks [55–58]. Because institutional culture varies and is affected by a variety of factors, including the size of the institution, its physical location, and the composition of the members who make up the institution, we did not aim to delineate the exact qualities of the institutional culture. Instead, we assessed the relative strength of the institutional culture, over other potential theoretical explanations at the ego-alter level, for the existence of social ties. If institutional culture is a significant predictor of the existence of social ties above and beyond other possible explanations, such as attractiveness or homophily, we would expect to find different predictors for the existence of social ties in different CCRCs, suggesting that no two CCRCs are alike.

In addition to the institutional culture, which can only be measured at the network level, we examined four sets of variables to assess the role of attractiveness and homophily at the dyadic level. Specifically, we assessed demographic characteristics (age, gender, education, tenure), health indicators (subjective health, number of chronic conditions, activities of daily living), social position (subjective social status, loneliness), and physical location indicators (wing, floor, and room proximity).

For the attractiveness hypothesis, we tested whether certain characteristics make the individual more attractive (i.e., have a higher number of people naming them as a significant contact). Because we expected younger residents to be seen as more attractive, given past research that shows that ageism hampers older adults’ social interactions [59], we examined age as an attractive factor. In line with past research that finds women to have larger networks [60], we expected women to be seen as more attractive socially. Tenure (length of stay in the CCRC) was also seen as a potential predictor of the existence of social ties, based on past research that identifies a significant association between tenure and network size [61]. We also examined health indicators, based on research showing that health status is a major determinant of close social ties [62], and social position as potential qualities that could make the formation of close ties more likely [63].

For the homophily hypothesis, we tested whether individuals who have characteristics in common were more likely to have strong social ties with one another. We did this by examining whether existing social ties (such as those based on gender or age difference) were randomly selected from the pool of all possible ties. In line with the previous research elaborated above, we expected individuals who share similar demographic characteristics [31, 32], health characteristics [64], and/or social position indicators [65] to be more likely to form close social ties to each other. Consistent with past research [39–41], physical location characteristics, including CCRC’s institutional culture, wing proximity, floor proximity, and room proximity were examined as potential predictors of the presence of close ties (i.e., a type of homophily). It is important to note that some aspects of our hypotheses were not as grounded in past research as others, as not all variables have been previously examined in relation to the existence of social ties among older adults. However, we decided to examine a relatively large number of potential predictors for the existence of social ties, given the limited research on the topic to date.

## Design and methods

### The sample and procedure

All residents in four CCRCs (designated as AG, MF, BY, and MJ in the study) in Israel were approached. All of the residents were eligible to participate, provided they spoke Hebrew or English and did not have a diagnosis of dementia as indicated in their medical records. This information was directly conveyed by the social worker in charge of the setting, as the researchers had no access to respondents’ medical records. Interviews were conducted face-to-face by trained research assistants. Most interviews occurred in the respondent’s room in the CCRC. The interviews were conducted between November 2016 and October 2017; in each setting, the interviews were conducted over a period of about three to four months. Potential respondents received information about the study in writing and through oral presentations. Respondents were able to opt out of the study at any time and there were no sanctions associated with lack of participation. The study was approved by the ethics committee of the school of social work at Bar Ilan University. All participants signed an informed consent.

The response rate was 60% in AG, 38% in MF, 31% in BY, and 36% in MJ. Of the non-responders (56% in AG, 53% in MF, 54% in BY, 70% in MJ), the main reasons were physical illness, cognitive impairment, or death. With the exception of MJ, the mean in-degree (the number of individuals in the CCRC who knew the person) of those who declined to participate was significantly higher than the mean in-degree of those who could not participate. This suggests that data are not missing at random. Data are available upon request.

### The settings

We selected the settings based on past research [59, 66] that stressed the importance of the CCRC’s features in shaping older residents’ social lives. We specifically aimed for geographic diversity by selecting settings from the center of the country and from Jerusalem. The center of the country tends to be characterized by more liberal views and greater wealth than Jerusalem, which is generally considered poorer and more conservative [67]. We also looked for variations in size (CCRCs ranging from 40 to 299 residents), socioeconomic status, and cultural background. This potentially allows for some generalization of the findings beyond the individual characteristics of a particular setting [68].

MF had 299 residents living in six adjacent wings. AG had 40 residents living in a five-story building. BY has two connected wings spread over four floors; at the time of this study, BY had 162 residents. Finally, MJ had 89 residents living in a five-story building. Other information about the specific characteristics of the four CCRCs can be found in a previous publication based on these data [14].

### Measures

#### Demographic characteristics

Age, gender, years of education, and tenure were gathered through self-report.

#### Health indicators

Medical status was assessed as the number of chronic conditions reported by the respondent (e.g., diabetes, hypertension, heart condition, stroke, arthritis; range 0–6). A higher score indicated more physical problems.

Overall functioning was assessed using an Activities of Daily Living (ADL) scale (e.g., requiring assistance in showering, dressing, or transfer; range 0–6), with a yes/no response format [69]. A higher score indicated greater impairment.

Subjective health was assessed using a single item, which asked respondents to rate their health on a five-point scale (5 = excellent; 1 = very poor).

#### Social position

The MacArthur Scale of Subjective Social Status is a 10-rung ladder measuring subjective socioeconomic standing. Participants were asked to mark the rung that best represents their social position within their community. On the top rung are the richest and best educated individuals; the poorest and least educated are at the bottom of the ladder [70, 71].

- Loneliness was assessed using the short R-UCLA Scale [72, 73], one of the most widely used scales of loneliness. The measure includes three questions (“How often do you feel you lack companionship?”; “How often do you feel left out of social activities?

” and “How often do you feel isolated from others?”), rated on a three-point scale (recoded as 1 = hardly ever or never; 3 = often). A mean score was calculated, with a higher overall score representing greater loneliness (range 1–3) (α = .83).

#### Physical location indicators

In addition to the CCRC setting, a defined location, which represents the institutional culture as an aggregate, we obtained information about the wing number, floor number, and room number of each of the respondents. For each respondent, we calculated a room distance (room proximity), a wing distance (wing proximity; for the two settings that had wings), and a floor distance (floor proximity) in relation to the location of all other residents. This information was used to calculate geographic homophily (physical proximity) in the ego network.

#### Constructing the social networks

Each respondent received a list of names of all individuals receiving services in the respective CCRC. All names appeared on the list, unrelated to whether or not these individuals participated in the study. Respondents were asked to indicate whether or not they were familiar with each of the CCRC residents. For each person on the list, respondents were asked the following question: “How likely are you to share your thoughts and secrets with this person?” The responses to this question were used to construct the social network of close social ties. The answers were rated on a five-point scale and dichotomized in this study to represent greater likelihood (4–5) or lower likelihood (1–3) of sharing thoughts and secrets. A directed link was portrayed only between individuals who reported ties at a level of 4 or higher. For instance, if resident A ranked the probability of sharing thoughts and secrets with resident B as 4 or 5, we included a directed link from A to B. But, if A ranked the probability of sharing thoughts and secrets as 3 or lower or did not rank it at all, we did not include a link from A to B. A link from B to A could still be present, provided B ranked the probability of sharing thoughts and secrets with A as 4 or higher.

#### Analysis

Our study used social network analysis methodology. Social networks are formally defined as a set of nodes (representing network members) connected by one or several types of relations, or edges [74]. Analysis was conducted using R [75] and the igraph package [76]. Analysis was restricted to those who participated in the survey. To test the study hypotheses, we followed several stages:

The attractiveness hypothesis was tested using correlations between the ego’s in-degree and all relevant attributes. This analysis was conducted within each setting to examine consistency across settings.

The homophily hypothesis was examined by comparing the median value of existing ties (e.g., with regard to age or gender) to that of all possible social ties as if they were randomly formed. A significant value indicates that tie formation based on gender, age, or any other attribute is significantly different from what would be expected if the social ties were made at random. Below is an example of the analysis, using age as an attribute of interest:

We define *Δage*_*ij*_ *≡ |age*_*i*_*−age*_*j*_*|* as the age difference between persons *i* and *j*. The link indicator *link*_*ij*_ indicates whether person *i* shares thoughts and secrets with person *j* (*link*_*ij*_ = 1) or not (*link*_*ij*_ = 0). We note that *link*_*ij*_ is a directed measure, implying that *link*_*ij*_ and *link*_*ji*_ are not necessarily equal. We then compare the median value of *Δage*_*ij*_ of the entire population to *Δage*_*ij*_ of existing links (*link*_*ij*_ = 1). A significant result indicates that a link is more likely to be formed when homophily exists (i.e., the connection between *i* and *j* is non-random) [77]. To test this set of hypotheses, we conducted the Mann-Whitney-Wilcoxson test, a nonparametric test to compare the median of a sample to the population. The Mann-Whitney test is used as an alternative to the t-test when the sample and population distributions are not normally distributed. A similar analysis was conducted for all attributes examined in this study (demographic data, health indicators, social position, and physical distance indicators). This analysis was conducted within each setting to examine consistency across settings.

**The role of the institutional culture.** We ran link prediction models with random forests (RFs) as a summative attempt to identify the various motivations behind social tie formation: the attractiveness of certain attributes, homophily versus the institutional culture (e.g., homophily at the network level). Link prediction predicts the likelihood of specific ties (links) in a given network [78]. Prediction can be based on the structure of the network or on the characteristics of the social nodes and ties. Random forest is an ensemble machine-learning method of classification using decision trees that produces an accurate and stable prediction model [78]. RF is often used as a mechanism for link prediction (see [79–81]). An important byproduct of RF is a variable-importance measure that ranks variables according to their importance in the prediction task. Following the algorithm in [82], we used the Conditional Inference (CI) RF model, with 100 trees.

Link prediction is originally a prediction problem; RFs are designed as a prediction algorithm with limited ability to explain the causal relationship between the predictors and the predicted value (see [83, 84]). However, in this analysis we took a reverse-engineering approach and focused on the classification process, rather than on the classification output. To that end, we constructed a link prediction problem based on the characteristics of the social nodes and ties and examined the variable-importance score of various attributes as potential predictors of the formation of ties.

The reason for choosing RF rather than other alternative link prediction classifiers, such as Lasso regression, or the logistic model, stems from the properties of the classification outcome. Specifically, while other classifiers might increase the link prediction accuracy, they lack the unbiased variable-importance scores that are outputted by RF.

To test the validity of our model, we divided the sample into training (60%) and validation (40%) samples. We ran the model on the training set and checked its accuracy on the validation set. Because of multicollinearity between attractiveness indicators and difference (homophily) indicators, we present two separate models: one to examine the role of attractiveness (on top of location indicators) and one to examine the role of homophily (on top of location indicators).

## Results

### The sample

Table 1 presents the demographic characteristics of the participants in each of the four settings. The average age of respondents was between 79.7 (SD = 25.8) (in MF) and 86.7 (SD = 5.7) (in BY). The majority of respondents in all four settings were female (ranging from 69% in MJ to 84% in MF). The average years of education ranged substantially: the average number of years of education in BY was 9.5 (SD = 5.6) and in AG 15.4 (SD = 4.7). The number of years in the CCRC was highest in AG (7.4 years; SD = 5.5) and BY (7.4 years; SD = 8.2) and lowest in MJ (3.3 years; SD = 3.0).

**Table 1:** Sample characteristics per Continuing Care Retirement Community (CCRC) setting.

Sample characteristics	AG (*N* = 23)	BY (*N* = 55)	MF (*N* = 115)	MJ (*N* = 36)
**Geographic location**	Jerusalem	Center	Center	Jerusalem
**Overall size**	40	162	299	89
***Demographic characteristics***				
**Age (M[SD])**	82.8(6.9)	86.7(5.7)	79.7(25.8)	84.3(9.3)
**Women (N,%)**	18(78%)	42(76%)	97(84%)	25(69%)
**Education in years (M[SD])**	15.4(4.7)	12.7(3.3)	13.6(4.2)	9.9(5.6)
**Tenure (years in the CCRC) (M[SD])**	7.4(5.5)	7.4(8.2)	6.5(7.1)	3.3(3.0)
***Health status***				
**Medical status (M[SD]) (0–6)**	1.3(1.2)	1.2(1.1)	1.3(1.1)	1.8(1.5)
**Overall functioning (M[SD]) (0–6)**	1.0(2.1)	1.6(.1)	.9(1.9)	1.5(.1)
**Subjective health (1–5)**	2.9(1.1)	2.9(1.0	2.6(.1)	2.6(1.0)
***Social position***				
**Subjective social status (M[SD]) (0–10)**	7.5(1.6)	7.6(1.5)	7.6(1.7)	7.2(2.0)
**Loneliness (M[SD]) (1–3)**	1.6(.7)	1.3(.4)	1.5(.6)	1.5(.6)

M = mean, SD = standard deviation; AG, BY, MF, MJ stand for the four different CCRCs.

Table 2 presents the social network characteristics of the sample. Network density represents the number of actual ties out of all possible ties. It ranges from 0 (no connections at all) to 1 (all possible connections are established). The lowest density was in MJ (.01); the highest was in AG (.09). Both represent a very low density, as only 9 out of 100 possible ties were identified as close ties in AG and 1 out of 100 ties were identified in MJ. Network components represent the proportion of the network that includes a path between each pair of individuals. The number of components ranges substantially from 28 in the largest CCRC (MF) to 1 in the smallest (BY). In-degree represents the number of people who nominated the respondent as a close tie; out-degree represents the number of people nominated by the respondent as close ties. The average in-degree and out-degree was 2.17 in AG, 3.44 in BY, 1.52 in MF, and .78 in MJ.

**Table 2:** Network characteristics.

Setting	Size	Density	Components	Mean in-degree	Standard deviation in-degree	Mean out-degree	Standard deviation out-degree
**AG**	40	0.09	3	2.17	2.14	2.17	2.68
**BY**	162	0.07	1	3.44	2.16	3.44	4.87
**MF**	299	0.01	28	1.52	1.63	1.52	3.36
**MJ**	89	0.03	14	0.78	0.91	0.78	1.29

Density- Number of actual ties out of all possible ties

Components- A proportion of the network that includes a path between each pair of individuals

In-degree- Number of people who nominated the respondent as a close tie

Out-degree- Number of people nominated by the respondent as close ties

AG, BY, MF, MJ stand for the four different CCRCs

### Testing the attractiveness hypothesis

Table 3 outlines the correlations of the in-degree with the various attributes per setting. Both the size and direction of the correlations were inconsistent across settings, providing limited support to the attractiveness hypothesis. In AG, none of the variables used to test the attractiveness hypothesis was significantly associated with the number of in-coming ties (more individuals nominating the ego as a close social tie). In BY, lower age, lower tenure, and lower levels of loneliness were significantly associated with number of in-coming ties. In MF, higher age was associated with more in-coming ties. In MJ, women were more likely to have more in-coming ties.

**Table 3:** Correlations of In-degree and attributes in each of the centers.

Sample characteristics	AG (*N* = 23)		BY (*N* = 55)		MF (*N* = 115)		MJ (*N* = 36)	
	Attractiveness estimate	p-value	Attractiveness estimate	p-value	Attractiveness estimate	p-value	Attractiveness estimate	p-value
***Demographic characteristics***								
**Age**	.19	0.41	-0.35	0.01	.20	0.03	-0.26	0.15
**Women**	.25	0.25	-0.25	0.09	-.09	0.32	0.36	0.04
**Education in years**	.07	0.77	0.02	0.91	.12	0.22	0.15	0.45
**Tenure (years in the CCRC)**	.07	.74	-.32	0.03	.20	.06	.21	.28
***Health status***								
**Medical status (0–6)**	-.19	0.38	-0.05	0.73	.10	0.29	-0.14	0.45
**Overall functioning (0–6)**	.39	0.07	0.07	0.64	-.14	0.13	0.05	0.79
**Subjective health (1–5)**	.15	0.51	0.21	0.16	.18	0.06	0.04	0.82
***Social position***								
**Subjective social status (0–10)**	-.39	0.17	0.27	0.17	.10	0.36	0.39	0.11
**Loneliness (1–3)**	.19	0.38	-0.27	0.07	.05	0.57	-0.13	0.50

AG, BY, MF, MJ stand for the four different CCRCs; In-degree- Number of people who nominated the respondent as a close tie; Attractiveness is measured as a correlation between in-degree and each of the different attributes at the ego level.

### Testing the homophily hypothesis

Table 4 presents the homophily tests per attribute for each of the four CCRCs. Table 4 shows that homophily operationalized as wing proximity and floor proximity was a significant predictor of the existence of close ties among residents in three of the four settings, and room proximity was a significant predictor in two of the settings. Overall functioning was also a significant predictor in two of the settings.

**Table 4:** Homophily tests per attribute.

CCRC setting	AG		BY		MF		MJ	
	Homophily estimate	p-value	Homophily estimate	p-value	Homophily estimate	p-value	Homophily estimate	p-value
***Demographic characteristic***								
**Age**	2.11	0.06	4.21	0.09	-1.44	0.04	4.27	0.31
**Gender**	0.41	0.01	0	*(low variability)*	0.18	0.04	0.25	0.50
**Education in years**	0.94	0.38	-0.25	0.39	-0.59	0.13	0.33	0.50
**Tenure (years in the CCRC)**	2.64	0.04	6.87	0.04	-1.51	0.04	2.13	0.12
***Health indicators***								
**Medical status (0–6)**	0.12	0.29	0.5	0.17	-0.14	0.28	0.50	0.29
**Overall functioning (0–6)**	0.88	0.04	4.5	0.07	0.86	0.01	0.00	*(low variability)*
**Subjective health (1–5)**	1.00	0.001	0.75	0.07	-0.50	0.001	1.00	0.19
***Social position***								
**Subjective social status (0–10)**	-0.25	0.50	0.00	*(low variability)*	-0.06	0.42	3.50	0.50
**Loneliness (1–3)**	0.08	0.45	0.17	0.17	0.03	0.41	0.56	0.50
***Physical location indicators***								
**Wing proximity**	Not relevant		0.45	.005	3.62	.03	Not relevant	
**Floor proximity**	-0.13	0.57	-0.09	0.02	-0.74	0.03	-0.62	0.02
**Room proximity**	-1.38	0.28	2.16	0.74	-28.75	0.002	-1.99	0.06

AG, BY, MF, MJ stand for the four different CCRCs; Homophily is estimated as the median value of existing ties compared against all possible social ties as if they were randomly formed

The remaining variables were inconsistent across settings. Gender dissimilarity was associated with a greater likelihood of tie existence in AG and in MF, but not in the other two settings. Tenure dissimilarity was associated with a greater likelihood of tie existence in AG and BY, but tenure homophily was true for MF. In MF, age homophily was also associated with the existence of close social ties. Subjective health dissimilarity was significantly associated with the existence of close social ties in AG, but subjective health homophily was true for MF.

### Testing the role of the institutional culture

**Fig 1:**
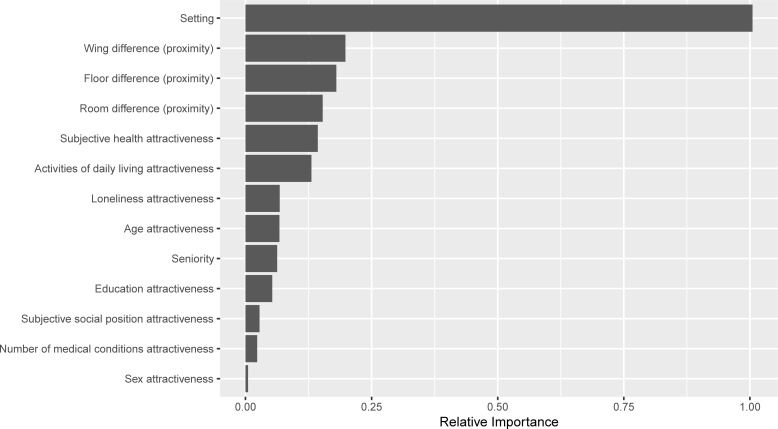
The link prediction model, using forest plot to examine location and attractiveness as predictors. Random Forest was used to obtain a variable-importance score of various attractiveness attributes and geographic proximity as potential predictors of the formation of ties. 1.00 represents the most important predictor of the presence of close ties between CCRC residents.

Fig 1 presents the relative importance of the different predictors, which is represented by the size of the bar. All four CCRCs were entered into the model simultaneously. The results show that the particular CCRC setting is the most important predictor (standardized as 1). This indicates that the networking culture differs between centers. Obviously, networks are structurally produced within each center and are thus limited to the particular CCRC, but this analysis shows that the most important split between individuals is determined by CCRC affiliation, suggesting that there is no other common denominator that can better explain the connections and that in each CCRC different strategies are used to facilitate connections between residents. The network strategy is most likely not defined by the individual members’ characteristics but rather by the local institutional culture, otherwise we would have seen the same strategy on average for all centers. This finding represents homophily based on affiliation with a particular institute. Next in importance is the physical location. Specifically, wing proximity and floor proximity are about four times less important than the specific setting, but almost two times more important than room proximity. The link prediction model demonstrated good accuracy of the validation model AUC = .95.

**Fig 2:**
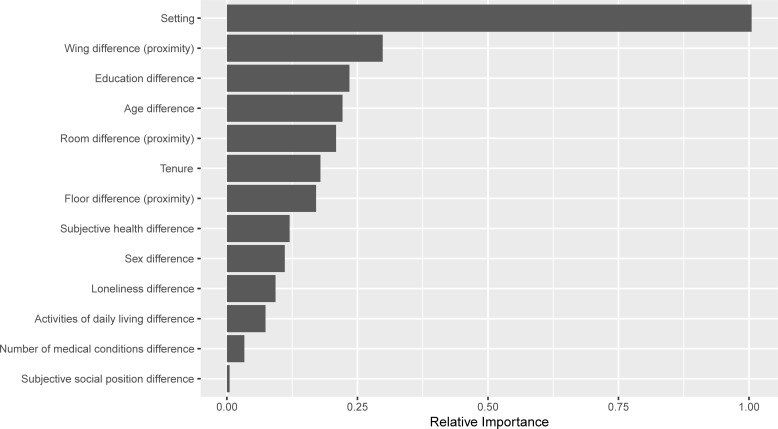
The link prediction model, using forest plot to examine location and homophily (noted as difference) as predictors. Random Forest was used to obtain a variable-importance score of various attractiveness attributes and geographic proximity as potential predictors of the formation of ties. 1.00 represents the most important predictor of the presence of close ties between CCRC residents.

When the RF model was conducted with regard to homophily, results were consistent, as detailed in Fig 2. The setting was the most important predictor, followed by wing proximity. These predictors were followed by education, age, floor proximity, and room proximity. This model had a validation accuracy of AUC = .98.

## Discussion

This study examined two possible bases for the existence of close ties between individuals [63]. The attractiveness theory suggests that certain attributes or qualities make an individual more attractive or popular than others, whereas the homophily theory argues that people form close relationships with those who are similar to them in certain characteristics. The institutional culture theory is a variant of the homophily theory, but it is tested at the entire level of the social network, rather than at the dyadic level of ego-alter relations. The institutional culture represents the aggregated collaborative values, attitudes, and practices common to a particular institution. It reflects homophily based on location as well as based on other potential variables. Some of these variables, such as age and education, were examined in this study, whereas others were not. Because the study was conducted in four different CCRCs, we had the opportunity to move beyond the specific particularities of a defined setting to examine the generalizability of the findings across settings. By doing so, we examined the potential place of the institutional culture (in this case, the CCRC setting) in shaping the social network [52].

The present study is unique in its ability to go beyond the particular CCRC. Previous research in the field of older adults’ social networks examined a single institution at a time [54]. Our study demonstrates that findings based on a single network may not be generalizable to other networks. This has theoretical, practical, and empirical implications. Theoretically, the findings support the institutional culture theory, which argues that the unique characteristics of a particular institution make certain ties more or less plausible. The findings emphasize the importance of the collective sum over the individuals who make up the group. We were unable to identify variables that consistently contributed to the existence of ties across all four settings: in each setting, different variables or combinations of variables explained the existence of ties. Practically, the findings suggest that no two CCRCs are the same and, as a result, interventions geared to foster close ties among participants should be tailored to the specific CCRC setting. Empirically, this study strikes a cautionary note by stressing the unique characteristics of the individual setting. As social network research in the field of gerontology has largely relied on findings from single settings [54], the present findings stress the need to go beyond a single institution to develop a comprehensive understanding of older adults’ social networks.

The only consistent determinants of the presence of close social ties were the physical location indicators. Specifically, the strongest predictor for the presence of close social ties was an affiliation with a particular CCRC. This means that the institutional culture differs between CCRCs. The fact that different variations of indicators were significant in the site-specific analyses that examined the attractiveness and homophily hypotheses further suggests that the institutional culture differs across these four sites. Otherwise we would have seen the same strategy on average in all centers [55].

Besides the unique characteristics of the particular CCRC, only wing proximity was identified as an important and consistent predictor of the presence of close social ties. This finding is consistent with past research conducted in CCRCs [42] as well as with research conducted in a variety of other populations [39–41]. This clearly supports the assertion that geographic proximity matters and potentially facilitates the creation of close social ties. The one CCRC exception in which geographic proximity did not play a role in determining the existence of close ties was AG, a small, 40-person CCRC. It is expected that in this type of setting, there is no real need to share a floor in order to form close relationships, as people already live in close proximity to each other.

The surveillance zone is defined as the space within the visual field of the home, in which older adults spend increasingly more time as their physical functioning deteriorates and their ability to explore areas farther away from their home declines [85]. Based on the results of the present study, the surveillance zone in the CCRC seems to be the wing. Our study demonstrated that the wing, rather than the floor or room, defines the area in which older adults form close social ties. This area is larger than one’s room or floor, but is still limited in its physical size. It fits nicely into Rowels’ (1981) definition of the surveillance zone [85]. Even in a setting that presents itself as a home—some of the CCRCs in this study literally incorporate the word “home” in their name—older adults’ relations seem to be confined to the wing, rather than to the entire space potentially offered to them by the setting.

Contrary to our expectations, demographic variables, health, and subjective social location did not play consistent roles in determining the presence of close ties between individuals. This finding is surprising given past research that emphasized the role of CCRC residents’ health in the presence of close social ties [62], as health is a scarce resource in the CCRC [9, 25]. Other demographic and subjective social position characteristics also did not have a consistent role in determining the presence of social ties. By examining four different networks, we demonstrate the uniqueness in each CCRC network that cannot necessarily be generalizable to other settings.

In reviewing these findings, several limitations should be noted. First, this is a cross-sectional design that does not allow for inferences about cause and effect. Second, the non-response to this study cannot be defined as missing at random, as those older adults who did not participate in this study because of cognitive problems and sickness were less known in the network (i.e., had a lower number of in-coming ties) [14]. Because the analysis of social network data benefits from obtaining data about the entire social network, the presence of missing values certainly suggests that results should be viewed with caution. Moreover, because all participants had to be above a certain age to join a CCRC and, thus, to participate in the study, limited variations with regard to age could potentially account for the fact that neither age attractiveness nor age homophily were consistent predictors in this study. Finally, although we examined a variety of explanations for the presence of close ties, alternative explanations not tested in this study might still be plausible. Other variables such as values or attitudes that were not explored in this study might possibly go beyond the institutional culture to account for the formation of close ties. However, at present there is no strong theory to suggest other potential predictors. Given the fact that only four CCRCs participated in this study, we cannot infer the particular characteristics of each setting that are responsible for the institutional culture. For this reason, we cannot say whether the size, the physical location, the socioeconomic status, or the cultural composition of the residents are responsible for the particular networking strategy employed in a particular CCRC. Finally, similar to past research conducted in CCRCs [42, 86], the findings should be considered within the overall context of the relatively low density of the social network, suggesting that very few individuals are likely to form close ties with others in their network.

### Implications

This study provides substantial insights into the social lives of older adults. Given the importance placed on close ties in old age, it is essential to further identify potential determinants responsible for the presence of such ties. Our findings point to the importance of the institutional culture. Specifically, our study shows that different attributes are associated with the presence of close ties in different CCRCs. On the one hand, this is quite logical, as we know that people in different settings appreciate different qualities and that different settings foster different social networks. On the other hand, this creates a challenge for health and long-term care professionals who are obligated to assess the particularities of their settings. The physical location defines the borders of close ties, more so than other characteristics examined in this study. Despite efforts to turn the CCRC into a home, the surveillance zone through which older adults explore the social world is likely confined to the CCRC wing. Long-term care administrators can encourage social activities at the wing level through the introduction of a wing lobby and dining hall, for instance.

## References

[1] Hawkley LC, Kocherginsky M. Transitions in loneliness among older adults: A 5-year follow-up in the National Social Life, Health, and Aging Project. Research on Aging. 2018;40(4):365–87. 10.1177/0164027517698965 29519211 PMC6355458

[2] Ayalon L, Shiovitz-Ezra S, Palgi Y. No place like home? Potential pathways to loneliness in older adults under the care of a live-in foreign home care worker. The Journal of Psychology. 2012;146(1–2):189–200. 10.1080/00223980.2011.574169 22303620

[3] Cattan M, White M, Bond J, Learmouth A. Preventing social isolation and loneliness among older people: a systematic review of health promotion interventions. Ageing & Society. 2005;25(1):41–67. 10.7748/nop.17.1.40.s11 27736564

[4] Dickens AP, Richards SH, Greaves CJ, Campbell JL. Interventions targeting social isolation in older people: a systematic review. BMC Public Health. 2011;11(1):647. 21843337 10.1186/1471-2458-11-647 PMC3170621

[5] Campbell N. Designing for social needs to support aging in place within continuing care retirement communities. Journal of Housing and the Built Environment. 2015;30(4):645–65.

[6] Iecovich E. The long-term care insurance law in Israel: present and future. Journal of Aging & Social Policy. 2012;24(1):77–92. 22239283 10.1080/08959420.2012.628892

[7] Gamliel T, Hazan H. The meaning of stigma: identity construction in two old-age institutions. Ageing & Society. 2006;26(3):355–71.

[8] Ayalon L. Intergenerational perspectives on autonomy following a transition to a continuing care retirement community. Research on Aging. 2015:0164027515575029. 10.1177/0164027515575029 25749736

[9] Ayalon L. Do not hear, see, or speak: views of older residents and their adult children about the nursing unit in the continuing care retirement community. International Psychogeriatrics. 2016;28(11):1867–77. 10.1017/S1041610216000788 27405736

[10] Bekhet AK, Zauszniewski JA, Nakhla WE. Reasons for relocation to retirement communities: A qualitative study. Western Journal of Nursing Research. 2009;31(4):462–79. 10.1177/0193945909332009 19246417

[11] Krout JA, Moen P, Holmes HH, Oggins J, Bowen N. Reasons for relocation to a continuing care retirement community. Journal of Applied Gerontology. 2002;21(2):236–56.

[12] Ayalon L, Greed O. A typology of new residents' adjustment to continuing care retirement communities. The Gerontologist. 2015. 10.1093/geront/gnu121 25614609

[13] Buys LR. Life in a retirement village: implications for contact with community and village friends. Gerontology. 2001;47(1):55 10.1159/000052771 11244293

[14] Ayalon L, Yahav I, Lesser O. From a bird's eye view: A social network perspective on older adults in adult day care centers and continuing care retirement communities. Innovation in Aging. 2018 10.1093/geroni/igy024 PMC6176959 30480144

[15] Ayalon L. Loneliness and anxiety about aging in adult day care centers and continuing care retirement communities. Innovation in Aging. 2018;2(2):igy021–igy. 10.1093/geroni/igy021 30480141 PMC6177038

[16] Crisp DA, Windsor TD, Butterworth P, Anstey KJ. Adapting to retirement community life: Changes in social networks and perceived loneliness. Journal of Relationships Research. 2015;6.

[17] Taylor HO, Wang Y, Morrow-Howell N. Loneliness in senior housing communities. Journal of Gerontological Social Work. 2018;61(6):623–39. 10.1080/01634372.2018.1478352 29791279 PMC6938254

[18] Herron RV, Funk L, Spencer D, Wrathall M. Assisted living facilities as sites of encounter: implications for older adults’ experiences of inclusion and exclusion. Ageing & Society. 2019:1–17.

[19] Elias SMS. Prevalence of loneliness, anxiety, and depression among older people living in long-term care: a review. International Journal Of Care Scholars. 2018;1(1):39–43.

[20] Carstensen LL. Social and emotional patterns in adulthood: support for socioemotional selectivity theory. Psychology and Aging. 1992;7(3):331 10.1037//0882-7974.7.3.331 1388852

[21] Anderson C, John OP, Keltner D, Kring AM. Who attains social status? Effects of personality and physical attractiveness in social groups. J Personality and SocialPsychology. 2001;81(1):116–32. Epub 2001/07/28. 10.1037//0022-3514.81.1.116 . 11474718

[22] Lukaszewski AW, Simmons ZL, Anderson C, Roney JR. The role of physical formidability in human social status allocation. Journal of Personality and Social Psychology. 2016;110(3):385 10.1037/pspi0000042 26653896

[23] Frevert TK, Walker LS. Physical attractiveness and social status. Sociology Compass. 2014;8(3):313–23.

[24] Feiler DC, Kleinbaum AM. Popularity, similarity, and the network extraversion bias. Psychological Science. 2015;26(5):593–603. 10.1177/0956797615569580 25838113

[25] Shippee TP. “But I am not moving”: Residents' perspectives on transitions within a continuing care retirement community. The Gerontologist. 2009;49(3):418–27. 10.1093/geront/gnp030 19372143

[26] Schafer MH. Health as status? Network relations and social structure in an American retirement community. Ageing & Society. 2016;36(1):79–105.

[27] Abbott KM, Bettger JP, Hampton KN, Kohler HP. The feasibility of measuring social networks among older adults in assisted living and dementia special care units. Dementia (London, England). 2015;14(2):199–219. Epub 2013/12/18. 10.1177/1471301213494524 . 24339099

[28] Casey A-NS, Low L-F, Jeon Y-H, Brodaty H. Residents perceptions of friendship and positive social networks within a nursing home. The Gerontologist. 2016;56(5):855–67. 10.1093/geront/gnv146 26603182

[29] Ayalon L. Perceptions of old age and aging in the continuing care retirement community. International Psychogeriatrics. 2015;27(04):611–20. 25391548 10.1017/S1041610214002415

[30] Marsden PV. Homogeneity in confiding relations. Social networks. 1988;10(1):57–76.

[31] McPherson M, Smith-Lovin L, Cook JM. Birds of a feather: Homophily in social networks. Annual Review of Sociology. 2001;27(1):415–44.

[32] Lazarsfeld PF, Merton RK. Friendship as a social process: A substantive and methodological analysis. Freedom and Control in Modern Society. 1954;18(1):18–66.

[33] Shrum W, Cheek NH Jr, MacD S. Friendship in school: Gender and racial homophily. Sociology of Education. 1988:227–39.

[34] Smith JA, McPherson M, Smith-Lovin L. Social distance in the United States: Sex, race, religion, age, and education homophily among confidants, 1985 to 2004. American Sociological Review. 2014;79(3):432–56.

[35] Hagestad GO, Uhlenberg P. The social separation of old and young: A root of ageism. Journal of Social Issues. 2005;61(2):343–60.

[36] Uhlenberg P, Gierveld JDJ. Age-segregation in later life: An examination of personal networks. Ageing & Society. 2004;24(1):5–28.

[37] Skopek J, Schulz F, Blossfeld H-P. Who contacts whom? Educational homophily in online mate selection. European Sociological Review. 2011;27(2):180–95. 10.1093/esr/jcp068

[38] Pickett KE, Pearl M. Multilevel analyses of neighbourhood socioeconomic context and health outcomes: a critical review. Journal of Epidemiology & Community Health. 2001;55(2):111–22. 11154250 10.1136/jech.55.2.111 PMC1731829

[39] Priest RF, Sawyer J. Proximity and peership: Bases of balance in interpersonal attraction. American Journal of Sociology. 1967;72(6):633–49. 10.1086/224400 6072037

[40] Schutte JG, Light JM. The relative importance of proximity and status for friendship choices in social hierarchies. Social Psychology. 1978:260–4.

[41] Tesch FE, Huston TL, Indenbaum EA. Attitude similarity, attraction, and physical proximity in a dynamic space 1. Journal of Applied Social Psychology. 1973;3(1):63–72.

[42] Schafer MH. On the locality of asymmetric close relations: spatial proximity and health differences in a senior community. The Journals of Gerontology Series B: Psychological Sciences and Social Sciences. 2015;70(1):100–10. 10.1093/geronb/gbu043 24814658

[43] Festinger L, Schachter S, Back K. Social pressures in informal groups; a study of human factors in housing. 1963 Palo Alto, CA, US: Stanford University Press.

[44] van den Berg YH, Cillessen AH. Peer status and classroom seating arrangements: A social relations analysis. Journal of Experimental Child Psychology. 2015;130:19–34. 10.1016/j.jecp.2014.09.007 25313926

[45] Kahn A, McGaughey TA. Distance and liking: When moving close produces increased liking. Sociometry. 1977:138–44.

[46] Katz AM, Hill R. Residential propinquity and marital selection: A review of theory, method, and fact. Marriage and Family Living. 1958;20(1):27–35.

[47] Ebbesen EB, Kjos GL, Konečni VJ. Spatial ecology: Its effects on the choice of friends and enemies. Journal of Experimental Social Psychology. 1976;12(6):505–18.

[48] Scott T, Mannion R, Davies H, Marshall M. The quantitative measurement of organizational culture in health care: a review of the available instruments. Health Services Research. 2003;38(3):923–45. 10.1111/1475-6773.00154 12822919 PMC1360923

[49] Parmelli E, Flodgren G, Beyer F, Baillie N, Schaafsma ME, Eccles MP. The effectiveness of strategies to change organisational culture to improve healthcare performance: a systematic review. Implementation Science. 2011;6(1):33. 21457579 10.1186/1748-5908-6-33 PMC3080823

[50] Fehr, Ernst and Hoff, Karla, Tastes, Castes, and Culture: The Influence of Society on Preferences. IZA Discussion Paper No. 5919. Available at SSRN: https://ssrn.com/abstract=1921970

[51] Gudykunst WB, Ting-Toomey S, Chua E. Culture and interpersonal communication. Thousand Oaks, CA: Sage Publications, Inc; 1988.

[52] Martin JN, Nakayama TK. Thinking dialectically about culture and communication. Communication Theory. 1999;9(1):1–25.

[53] Steensma HK, Marino L, Weaver KM, Dickson PH. The influence of national culture on the formation of technology alliances by entrepreneurial firms. Academy of Management Journal. 2000;43(5):951–73.

[54] Ayalon L, Levkovich I. A systematic review of research on social networks of older adults. The Gerontologist. 2018. 10.1093/geront/gnx218 29385450

[55] Emirbayer M, Goodwin J. Network analysis, culture, and the problem of agency. American Journal of Sociology. 1994;99(6):1411–54.

[56] Kim Y, Sohn D, Choi SM. Cultural difference in motivations for using social network sites: A comparative study of American and Korean college students. Computers in Human Behavior. 2011;27(1):365–72.

[57] Brass DJ, Galaskiewicz J, Greve HR, Tsai W. Taking stock of networks and organizations: A multilevel perspective. Academy of Management Journal. 2004;47(6):795–817.

[58] Schein EH. Culture: The missing concept in organization studies. Administrative Science Quarterly. 1996:229–40.

[59] Ayalon L. Perceptions of old age and aging in the continuing care retirement community. International Psychogeriatrics. 2015;27(4):611–20. 10.1017/S1041610214002415 25391548

[60] Antonucci TC, Akiyama H. An examination of sex differences in social support among older men and women. Sex Roles. 1987;17(11–12):737–49.

[61] Schafer MH. Structural advantages of good health in old age investigating the health-begets-position hypothesis with a full social network. Research on Aging. 2013;35(3):348–70.

[62] Schafer MH. Health and network centrality in a continuing care retirement community. The Journals of Gerontology Series B: Psychological Sciences and Social Sciences. 2011;66B(6):795–803. 10.1093/geronb/gbr112 21979938

[63] Papadopoulos F, Kitsak M, Serrano MÁ, Boguná M, Krioukov D. Popularity versus similarity in growing networks. Nature. 2012;489(7417):537 10.1038/nature11459 22972194

[64] Centola D. An experimental study of homophily in the adoption of health behavior. Science. 2011;334(6060):1269–72. 10.1126/science.1207055 22144624

[65] Cacioppo JT, Fowler JH, Christakis NA. Alone in the crowd: The structure and spread of loneliness in a large social network. Journal of Personality and Social Psychology. 2009;97(6):977 10.1037/a0016076 19968414 PMC2792572

[66] Ayalon L, Green V. Grief in the initial adjustment process to the continuing care retirement community. Journal of aging studies. 2012;26(4):394–400. 10.1016/j.jaging.2012.05.001 22939535

[67] Ram U. The globalization of Israel: McWorld in Tel Aviv, Jihad in Jerusalem: Routledge; 2013.

[68] Patton MQ. Qualitative research In Encyclopedia of Statistics in Behavioral Science (eds Everitt B. S. and Howell D. C.). Wiley Online Library; 2005 10.1002/0470013192.bsa514

[69] Lawton MP, Brody EM. Assessment of older people: self-maintaining and instrumental activities of daily living. The gerontologist. 1969;9(3_Part_1):179–86. 5349366

[70] Adler NE, Epel ES, Castellazzo G, Ickovics JR. Relationship of subjective and objective social status with psychological and physiological functioning: preliminary data in healthy white women. Health Psychology. 2000;19(6):586–92. 10.1037//0278-6133.19.6.586 . 11129362

[71] Singh-Manoux A, Marmot MG, Adler NE. Does subjective social status predict health and change in health status better than objective status? 10.1097/01.psy.0000188434.52941.a0 Psychosomatic Medicine. 2005;67(6):855–61. 16314589

[72] Russell D, Peplau LA, Cutrona CE. The revised UCLA Loneliness Scale: concurrent and discriminant validity evidence. Journal of Personality and Social Psychology. 1980;39(3):472–80. Epub 1980/09/01. 10.1037//0022-3514.39.3.472 7431205

[73] Hughes ME, Waite LJ, Hawkley LC, Cacioppo JT. A Short Scale for Measuring loneliness in large surveys results from two population-based studies. Research on Aging. 2004;26(6):655–72. 10.1177/0164027504268574 18504506 PMC2394670

[74] Wasserman S, Faust K. Social network analysis: Methods and applications: Cambridge, UK: Cambridge university press; 1994.

[75] R Development Core Team 2009: R: A language and environment for statistical computing. Vienna, Austria Internet: http://www.R-project.org.2012.

[76] Csardi G, Nepusz T. The igraph software package for complex network research. International Journal of Complex Systems. 2006;1695(5):1–9.

[77] Athanasiou R, Yoshioka GA. The spatial character of friendship formation. Environment and behavior. 1973;5(1):43.

[78] Liben‐Nowell D, Kleinberg J. The link‐prediction problem for social networks. Journal of the American Society for Information Science and Technology. 2007;58(7):1019–31.

[79] Zhang Y., Pang J. Distance and friendship: A distance-based model for link prediction in social networks In: Cheng R., Cui B., Zhang Z., Cai R., Xu J. (eds). Web Technologies and Applications. APWeb 2015. Lecture Notes in Computer Science, vol 9313 2015; Springer, Cham

[80] Caragea D, Bahirwani V, Aljandal W, Hsu WH, editors. Ontology-based link prediction in the livejournal social network. Eighth Symposium on Abstraction, Reformulation, and Approximation; 2009.

[81] Wang X, Sukthankar G, editors. Link prediction in multi-relational collaboration networks. Proceedings of the 2013 IEEE/ACM international conference on advances in social networks analysis and mining; 2013: ACM.

[82] Hothorn T, Hornik K, Zeileis A. Unbiased recursive partitioning: A conditional inference framework. Journal of Computational and Graphical statistics. 2006;15(3):651–74.

[83] Liaw A, Wiener M. Classification and regression by randomForest. R news. 2002;2(3):18–22.

[84] Guns R, Rousseau R. Recommending research collaborations using link prediction and random forest classifiers. Scientometrics. 2014;101(2):1461–73.

[85] Rowles GD. The surveillance zone as meaningful space for the aged. The Gerontologist. 1981;21(3):304–11. 10.1093/geront/21.3.304 7239259

[86] Casey A-NS, Low L-F, Jeon Y-H, Brodaty H. Residents perceptions of friendship and positive social networks within a nursing home. The Gerontologist. 2015;56(5):855–67. 10.1093/geront/gnv146 26603182

